# Molecular characterization and comparative genomic analysis of *Acinetobacter baumannii* isolated from the community and the hospital: an epidemiological study in Segamat, Malaysia

**DOI:** 10.1099/mgen.0.000977

**Published:** 2023-04-05

**Authors:** Nazmul Hasan Muzahid, Md Hamed Hussain, Marie Andrea Laetitia Huët, Jacky Dwiyanto, Tin Tin Su, Daniel Reidpath, Faizah Mustapha, Qasim Ayub, Hock Siew Tan, Sadequr Rahman

**Affiliations:** ^1^​ School of Science, Monash University Malaysia, 47500, Bandar Sunway, Selangor Darul Ehsan, Malaysia; ^2^​ South East Asia Community Observatory (SEACO) and Global Public Health, Jeffrey Cheah School of Medicine and Health Sciences, Monash University Malaysia, Bandar Sunway, 47500, Subang Jaya, Selangor, Malaysia; ^3^​ Department of Pathology, Hospital Segamat, Jalan Genuang, Bandar Putra, 85000, Segamat, Johor, Malaysia; ^4^​ Monash University Malaysia Genomics Facility, 47500, Bandar Sunway, Selangor Darul Ehsan, Malaysia; ^5^​ Tropical Medicine and Biology Multidisciplinary Platform, Monash University Malaysia, Bandar Sunway, 47500, Subang Jaya, Selangor, Malaysia

**Keywords:** *Acinetobacter baumannii*, community, hospital, faecal, comparative genomics, antimicrobial resistance, virulence, CRISPR–Cas, Malaysia

## Abstract

*
Acinetobacter baumannii* is a common cause of multidrug-resistant (MDR) nosocomial infections around the world. However, little is known about the persistence and dynamics of *A. baumannii* in a healthy community. This study investigated the role of the community as a prospective reservoir for *A. baumannii* and explored possible links between hospital and community isolates. A total of 12 independent *A. baumannii* strains were isolated from human faecal samples from the community in Segamat, Malaysia, in 2018 and 2019. Another 15 were obtained in 2020 from patients at the co-located tertiary public hospital. The antimicrobial resistance profile and biofilm formation ability were analysed, and the relatedness of community and hospital isolates was determined using whole-genome sequencing (WGS). Antibiotic profile analysis revealed that 12 out of 15 hospital isolates were MDR, but none of the community isolates were MDR. However, phylogenetic analysis based on single-nucleotide polymorphisms (SNPs) and a pangenome analysis of core genes showed clustering between four community and two hospital strains. Such clustering of strains from two different settings based on their genomes suggests that these strains could persist in both. WGS revealed 41 potential resistance genes on average in the hospital strains, but fewer (*n*=32) were detected in the community strains. In contrast, 68 virulence genes were commonly seen in strains from both sources. This study highlights the possible transmission threat to public health posed by virulent *A. baumannii* present in the gut of asymptomatic individuals in the community.

## Data Summary

All sequence data are available online. The assembled sequencing reads generated in the present study are publicly available at the National Center for Biotechnology Information (NCBI), BioProject Number: PRJNA851747 and PRJNA659865. The authors confirm that all supporting data, code and protocols have been provided within the article or through supplementary data files. All new multilocus sequence typing (MLST) sequences generated were deposited onto the respective databases available on PubMLST (https://pubmlst.org/organisms/Acinetobacter-baumannii). Additional *A. baumannii* genomes used in this study are available and can be downloaded from GenBank (accession numbers available in File S2).

**Impact Statement**

Studies on *Acinetobacter baumannii* isolated from healthy individuals in the community are quite rare. Hospital-derived isolates are used in the majority of studies currently available. We obtained *A. baumannii* isolates from the faeces of community members in Malaysia and contrasted them with isolates from local hospitals. According to our findings, low and transient carriage of *A. baumannii* was deduced in the gut of community individuals. Community isolates were similar to hospital isolates in terms of virulence determinants but not antibiotic resistance. Using core-genome single-nucleotide polymorphism (SNP), pangenome, phylogenetic and CRISPR array analyses from whole-genome sequencing, it was demonstrated that some hospital and community isolates clustered together in all of the analyses. Our findings highlight the significance of *A. baumannii* carriage in healthy persons as well as the potential transmission risk. If strains of *A. baumannii* present in the gut acquire antimicrobial resistance (AMR) genes, infections caused by these organisms may become difficult to treat.

## Introduction

Antimicrobial resistance (AMR) has been a global threat to public health and a leading cause of long-term hospitalization, morbidity, mortality and costs over the years [1]. Hospital settings were considered to be the primary reservoirs of infections caused by antimicrobial-resistant bacteria, but a more pressing concern now is the spread of antimicrobial-resistant bacteria within the community and the environment [2, 3]. Widespread use of antimicrobials is the principal cause of the development of AMR inside and outside of the hospital [4]. Studies have demonstrated that communities can act as a key reservoir for antimicrobial-resistant bacteria [5–7].

*Acinetobacter baumannii,* a lactose-non-fermenting Gram-negative pathogen, is one of the most commonly identified multidrug-resistant bacteria. Although it was formerly considered to be a low-category pathogen, it has now emerged as the primary cause of hospital and community-acquired infections [8]. *A. baumannii* is an opportunistic pathogen that belongs to the ESKAPE group categorized by the Infectious Diseases Society of America (IDSA) [9]. The World Health Organization (WHO) has designated this bacterium as a priority 1 critical pathogen since 2017 [10]. Three major global clones of *A. baumannii* (IC- I, IC- II and IC- III) emerged as high-risk pandemic lineages with significant persistence in hospital settings [11]. Multidrug resistance is frequently linked to isolates from these global clones [12, 13]. *A. baumannii* has been related to a wide range of human diseases, including ventilator-associated pneumonia, bloodstream, skin and urinary tract infections, and secondary meningitis [14].

A combination of mechanisms, including drug target modification, the production of hydrolyzing enzymes such as beta-lactamases, alteration of bacteria cell membrane permeability, increased expression of efflux pumps and altered topoisomerases can lead to antibiotic resistance in *A. baumannii* [1, 15]. In addition, the ability of *A. baumannii* to develop biofilms on a wide range of surfaces can be associated with its persistence in hospital settings and the emergence of recalcitrant and chronic infections [16].

*A. baumannii* has been isolated from diverse sources, including hospitalized and non-hospitalized individuals, different environmental sources and slaughtered animals, but the ecology outside hospitals is not well understood [9, 17–20]. The mechanisms of *A. baumannii* virulence are well characterized in different settings [17, 18]. Although significant genomic differences and differences in phenotypes associated with virulence have been reported between single community-acquired and hospital-acquired strains, data on a larger number of community and hospital isolates from one locality to corroborate these observations are still lacking [19]. Without triggering an infection, *A. baumannii* can also colonize the skin and respiratory system [17]. In hot and humid climates – notably in the Asia-Pacific region – *A. baumannii* has emerged as a cause of severe community-acquired infections [21–23]. Studies have found *Acinetobacter* spp. in the intestinal microbiota of healthy volunteers (12.2 %) [24]. Nevertheless, the origins of colonization and factors influencing it are still unknown [25].

This study aimed to explore the role of the community as a potential reservoir for *A. baumannii* and possible transmission between the community and the hospital. We characterized *A. baumannii* isolates from the community in Segamat, a small town in southern peninsular Malaysia, and compared them with isolates obtained from a local government hospital. A longitudinal study was also conducted to detect whether isolates are commensal or transient in the community. The AMR profile and biofilm-forming ability of *A. baumannii* isolates from the community and the hospital were compared. Whole-genome sequencing (WGS) was used to investigate virulence determinants, as well as genes linked with antibiotic resistance and the presence of CRISPR arrays, followed by single-nucleotide polymorphism (SNP) and pangenome-based phylogenetic correlation between these strains. This study sheds light on the relationship between hospital and community isolates from one district in Malaysia, contributing to a better understanding of the epidemiology and pathogenesis of *A. baumannii* in the community.

## Methods

### Study location and sampling

The study was conducted in collaboration with the South East Asia Community Observatory (SEACO), a community-based research platform in Segamat District, Johor, Malaysia [26]. The human faecal samples were sourced from recruited people as described in [27]. Briefly, fresh faecal samples were collected from participants between May and August 2018. Resampling was carried out from the same individuals and other household members in November 2019 to determine if the isolates are transient or commensal and their transmission between other family members. Hospital Segamat provided hospital isolates from June to October 2020. This is the only tertiary government hospital in Segamat District, Johor, Malaysia.

### Isolation and identification of *A. baumannii*

To isolate *A. baumannii *, approximately 1 g of faecal sample was suspended in 9 ml of buffered peptone water (Oxoid, UK) and vortexed. Following this, a 10-fold serial dilution was carried out using buffered peptone water. From each dilution, 100 µl was spread on Leeds Acinetobacter Agar (HiMedia, India) and subsequently plates were incubated at 37 °C for 24 h. The colony morphology and nature of the strains were observed and recorded. Three colonies with *A. baumannii* morphology were selected and identified from each potentially positive sample based on aerobic, Gram-negative, catalase-positive, oxidase-negative, nonmotile, nonfermenting coccobacilli nature [28].

PCR amplification of 16S rRNA gene fragments and subsequent sequencing was performed to confirm *Acinetobacter* spp. at the genus level. The 16S rRNA gene was targeted using the universal primers described in previous studies [29]. Bacterial DNA extraction for PCR was carried out by the boiling extraction method [30]. Details of the extraction method and 16S rRNA PCR reaction can be found in Text S1, Table S7-S9 (available in the online version of this article).

Species identification by phenotypic methods and 16S rRNA is insufficient for unambiguous identification of *A. baumannii* [31]. Thus, the detection of *A. baumannii* with species-specific PCR was performed based on [32]. The internal fragment of *gyrB* gene was targeted for PCR amplification to detect *A. baumannii* (detailed in Table S10 and S11). *A. baumannii* ATCC BAA 1605 (ATCC BAA-1605) was used as control.

### Antimicrobial resistance profiling

Antibiotic susceptibility of *A. baumannii* isolates was determined against 13 antibiotics using the Kirby–Bauer disc diffusion method. The different classes of antibiotics used were penicillin (piperacillin, PRL 100); ß-lactamase inhibitor combinations (piperacillin–tazobactam, TZP 110; ampicillin–sulbactam, SAM 20); third- and fourth-generation cephalosporin (ceftazidime, CAZ 30; cefotaxime, CTX 30; cefepime, FEP 30); carbapenem (imipenem, IPM 10; meropenem MEM, 10); aminoglycoside (gentamicin, CN 10; amikacin AK 30); fluoroquinolone (ciprofloxacin, CIP 5) and tetracycline (TET 30). For colistin and polymyxin B, the broth microdilution method was used. Results were interpreted based on the Clinical and Laboratory Standard Institute (CLSI) guidelines [33]. *A. baumannii* ATCC BAA 1605 (ATCC BAA-1605) and *Escherichia coli* ATCC BAA 2325 (ATCC BAA-2523) with known resistance patterns were purchased from the American Type Culture Collection (ATCC, USA) and used in the study as controls. Three biological and technical replicates were used for this profiling.

### Biofilm formation analysis

Biofilm formation by each isolate was detected using crystal violet (CV) and XTT assays. Biofilm production and quantification using CV assay were performed according to [34] and the XTT assay was performed according to [35] with slight modifications. The results were interpreted according to the criteria suggested in [36]. Detailed descriptions of the XTT and CV assay can be found in Text S1.

### WGS

The total genomic DNA of *A. baumannii* isolates was extracted using the Wizard genomic DNA Extraction kit (Promega, USA) according to the manufacturer’s instructions. Extracted DNA quality and concentration were assessed using a Nanodrop bioanalyser spectrophotometer (Thermo Scientific, USA). Illumina sequencing libraries were prepared using the Nextera XT DNA Preparation Kit (Illumina, USA). All isolates were sequenced using the MiSeq Reagent kit v3 with a 2×251 bp paired-end read configuration.

Illumina reads were processed using Trimmomatic with the following parameters: PE, ILLUMINACLIP: adapters/NexteraPE.fa:2 : 30 : 10 : 8, LEADING:3, TRAILING:3, SLIDINGWINDOW:5 : 20, MINLEN:35 [37]. For each sequenced strain, the trimmed paired-end reads were then *de novo* assembled using SPAdes 3.15.2 [38]. Functional annotation was performed using Prokka 1.13 [39]. The MLST Oxford scheme was performed by targeting seven housekeeping genes (*glt*A, *gdhB*, *gyrB*, *gpi*, *cpn60*, *recA* and *rpoD*) from the assembled WGS sequences to obtain the allele profile and sequence types (STs) using the MLST 2.0 online tool at the Center for Genomic Epidemiology (CGE) (https://cge.cbs.dtu.dk/services/MLST/) [40]. Subsequently, goeBURST analysis (http://eburst.mlst.net/) was carried out to determine the clonal complexes present in our isolates and *A. baumannii* isolates in the PubMLST database (https://pubmlst.org/). AMR genes were identified by Abricate v1.0.1 (https://github.com/tseemann/abricate), using the Comprehensive Antibiotic Resistance Database (CARD) [41] and ResFinder [42]. Virulence-associated genes were identified using the virulence factor database (VFDB4) [43]. Gene content matrices were obtained using Roary 3.13.0 [44], with a minimum of 90 % identity between coding sequences (CDSs) required for a gene to belong to the same family. The CRISPR–Cas array was detected using CRISPRCasFinder 4.2.20 [45]. The number of CRISPR arrays in each genome with an evidence level ≥2 was counted and allocated to the genome. Mobile genetic elements from each isolate were detected using Mobile Element Finder v1.0.3 at the CGEserver. Single-nucleotide variants (SNVs) were detected using the SNIPPY v4.6.0 variant calling tool with multi-SNIPPY default parameters [46]. SNP sites were used to identify core genomic SNPs [47]. Genealogies Unbiased By recomBinations In Nucleotide Sequences (Gubbins) with default parameters was used to detect and analyse SNPs likely introduced simultaneously during a homologous recombination event [48]. Phylogenetic trees were built using Gubbins based on the alignment of the non-recombinant SNPs obtained and using a maximum-likelihood (ML) phylogeny inferred from the alignment of these SNPs. Phylogenetic trees were constructed using FastTree [49]. Phylogenetic clusters (*n*≥2) were identified using TreeCluster with a bootstrap threshold of 90 % and a genetic distance of 0.045 [50]. The online tool iTOL V6 was used to annotate and visualize the phylogenetic trees [51].

## Results

### Phenotypic characterization of *A. baumannii* isolates from the Segamat community and hospital

A total of 233 human faecal samples from 110 households in Segamat, Malaysia were tested in 2018 for the presence of *A. baumannii *. After screening, only nine *A *. *baumannii* were isolated. In 2019, resampling was carried out for all of these carriers and community members, where 3 *A. baumannii* isolates were detected from 126 faecal samples and all of these were from new individuals (2.4 %) and households. Based on our resampling data in 2019, none of the individuals carried *A. baumannii* for a year. Thus, overall 12 community strains were characterized.

In addition, 15 *A *. *baumannii* isolates were provided by Hospital Segamat from hospitalized patients. These isolates were obtained from blood culture (*n*=5), tracheal aspirates (*n*=7), urine (*n*=1) and sputum (*n*=1) assessments. One of the isolates lacked information concerning the isolation source. Detailed information on community and hospital isolates can be found in Tables S1 and S2.

#### Substantial differences in antimicrobial susceptibility profile

**Fig. 1.**
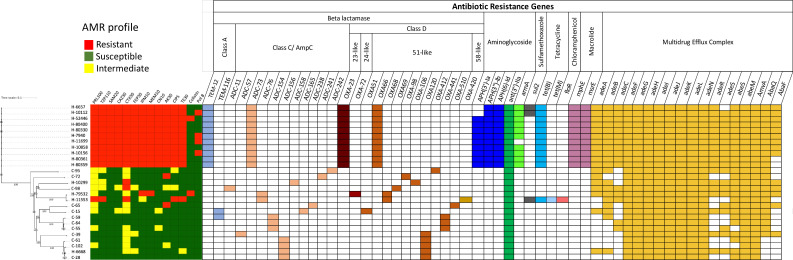
Antibiotic susceptibility profile and presence of resistance genes from whole-genome sequences of independent *A. baumannii* isolated from the Segamat community and hospital. Fourteen antibiotics were tested: PRL100, piperacillin 100 µg; TZP110, piperacillin–tazobactam 110 µg; SAM20, ampicillin–sulbactam; CAZ30, ceftazidime 30 µg; CTX30, cefotaxime 30 µg; FEP30, cefepime 30 µg; IPM10, imipenem 10 µg; MEM10, meropenem 10 µg; CN10, gentamicin 10 µg; AK30, amikacin 30 µg; CIP5, ciprofloxacin 5 µg; colistin (≥4 µg ml^−1^) and polymixin B (≥4 µg ml^−1^). The isolates were organized here along with a phylogenetic tree (left) constructed by whole-genome SNP analysis. In this study, isolate names with ‘H’ and ‘C’ represent the hospital and community isolates, respectively. The presence of genes in an isolate is specified by a coloured rectangle, with different genes coloured differently according to the classes to which they confer resistance. The absence of genes is represented by white rectangles.

The analysis of the antibiotic susceptibility profile of *A. baumannii* community and hospital isolates revealed considerable differences. Out of 15 hospital isolates examined, 12 (80 %) were classified as MDR and were resistant to cephalosporin, carbapenems, fluoroquinolones, aminoglycosides, tetracycline and β-lactam combination agents (Fig. 1). In addition, 2/15 and 4/15 of the hospital isolates showed resistance to colistin and polymyxin B, respectively (Fig. 1). In contrast, only three (25  %) of the community isolates showed resistance to one or two antibiotics and none were MDR.

These data showed that multidrug resistance was more common in the *A. baumannii* hospital isolates than the community strains (Fisher’s exact test *P*-value <0.0001).

#### Similar biofilm formation ability

Crystal violet (CV) and XTT assays were used to measure the biofilm biomass and metabolic activity of *A. baumannii* isolates. Both community and hospital isolates had variable abilities to form biofilms and the results varied depending on the assay used. However, the hospital strains had somewhat higher biofilm-producing ability compared to the community ones (Fig. S1), but the difference was not statistically significant. (chi-square test, *P*-value >0.05).

To determine the association between biofilm-forming ability and antibiotic resistance, statistical analysis (chi-square test) was carried out. However, no significant association was found between biofilm-forming ability and antibiotic resistance among these *A. baumannii* strains (*P*-value >0.05).

### Genomic characterization of *A. baumannii* isolates from the Segamat community and hospital shows relatedness

All 27 *A *. *baumannii* strains were subjected to WGS. Selected features of the sequenced *A. baumannii* isolates are shown in File S2 (Genomic features). The assembled draft genomes of these strains showed an average cumulative length of 3.6–3.8 Mb and 39 % GC content, consistent with the genome assembly of most published *A. baumannii* isolates in the GenBank database. The newly sequenced isolates from Segamat, Malaysia reported here showed more than 97 % pairwise average nucleotide identity (ANI) with the *A. baumannii* reference strain AC30.

#### MLST

STs of community and hospital *A. baumannii* isolates were determined using the MLST Oxford scheme from WGS-assembled sequences. The analysis identified 19 different STs from the 27 hospital and community isolates. Among them, nine STs (ST1930, ST2230, ST2232, ST2234 and ST2236 from the community and ST2237, ST2238 and ST2241 from the hospital) are being described for the first time in this study and were deposited in the PubMLST database (Table S3). The existing STs detected here are ST128, ST231, ST503, ST1463 and ST1912 from the community and ST208, ST447, ST547, ST642 and ST684 from the hospital. The remaining isolates were assigned new STs. No identical STs were detected between the community and hospital strains. ST208 was the predominant ST, comprising (46.6 %, 7/15) of the hospital isolates.

Based on the goeBURST analysis, eight clonal complexes (CCs) were detected, summarized in Table S5. They are CC208, CC231, CC474, CC953, CC642, CC1108, CC1171 and CC1178. ST208, ST547 and ST684 belong to the globally distributed clonal complex CC208 (previously known as CC92), which corresponds to IC2 (international clone 2) (Fig. S2). CC208 has been identified as a major epidemic clonal complex of carbapenem-resistant *A. baumannii* [52]. All CC208 isolates were obtained from the hospital. Interestingly, one community isolate, C-98 (ST231), belonged to CC231 that clustered with the previously identified international clonal lineage IC1.

#### SNV analysis

**Fig. 2.**
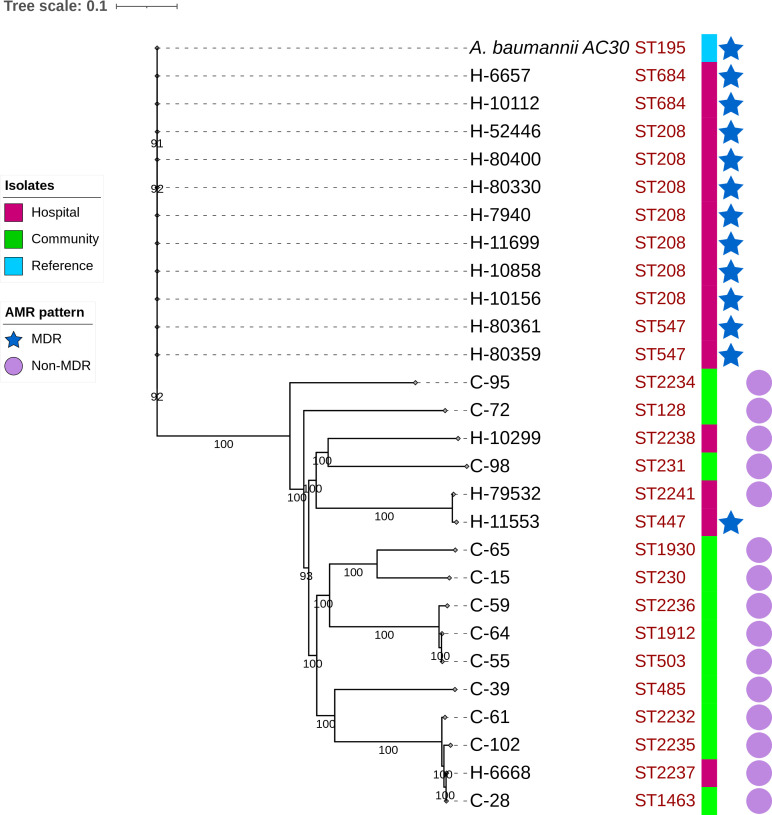
Phylogenetic tree using the maximum-likelihood method based on the core-genome SNP alignment of 27 sequenced community and hospital strains along with *A. baumannii* strain AC30 as a reference. Isolates are colour coded as purple and green for hospital and community isolates, respectively, and STs for each isolate are shown. The isolates’ sources and AMR patterns are displayed at the right and are represented by coloured squares, an asterisk for MDR isolates and circles for non-MDR, respectively. Bootstrap values are shown on each node.

The variation between the genomes of the current study, along with a reference strain *A. baumannii* AC30, was assessed. Considerable differences between *A. baumannii* MDR and sensitive isolates were observed. Against the reference strain, 11 MDR hospital isolates showed less nucleotide variation than the other 16 strains (Table S5). The number of nucleotide insertions and deletions was also different within the MDR and non-MDR classes. Total SNPs detected ranged from 447 (H-11699) to 39 120 (H-79532). A phylogenetic tree was constructed based on the core-genome SNP alignment (Fig. 2). From the phylogeny, two clusters were observed between hospital and community strains. In one cluster, three community strains were located in one clade with one hospital strain (C-28, C-102, C-61 and H-6668). The number of SNPs in these isolates was 36 447, 36 205, 36 572 and 37 320, respectively. The other cluster was observed between C-98 and H-10299, with 37 745 and 37 773 SNPs, respectively. Only H-11533 was MDR out of these hospital strains, clustering with the non-MDR strain H-79532.

#### Antibiotic resistance-related genes

The variable profile of antibiotic resistance identified among the strains led us to analyse the known resistance-associated genes in the sequenced genomes. These included genes encoding intrinsic and acquired β-lactamases, genes that confer resistance to aminoglycosides, fluoroquinolones, chloramphenicol, macrolides and tetracycline, as well as efflux pump-associated genes, even though not all may necessarily be required for resistance (Fig. 1).

A total of 55 antibiotic resistance genes were detected across all 27 isolates. Three types of β-lactamase genes encoding enzymes for class A, class C and class D β-lactamase superfamilies were detected in our isolates. The intrinsic AmpC β-lactamase (*bla*_ADC_) and OXA-51 serine-type oxacillinase (*bla*_OXA-51_) genes were found in all *A. baumannii* isolates (*n*=27) [53]. However, translations of the sequence revealed different STs corresponding to different protein clades for products of both these genes (Figs S3 and S4) [54]. Such differences in sequence may be allied with the differences in the phenotypes seen. Further, the OXA24 type gene (*bla*_OXA72_) and OXA58 type gene (*bla*_OXA-420_), which confer carbapenem resistance, were found in hospital isolates H-79532 and H-11553, respectively. Another widely distributed carbapenem-resistance gene, *bla*_OXA23_, and an extended-spectrum β-lactamase (ESBL) gene (TEM-12) were only detected in hospital strains (*n*=11/15). Two community isolates also harboured ESBL containing the gene for TEM-116.

The mechanisms by which *A. baumannii* develops resistance to aminoglycoside agents are varied, but they almost always involve the production of aminoglycoside-modifying enzymes. These enzymes can be categorized as aminoglycoside acetyltransferases (AAC), aminoglycoside phosphotransferases (APH), and/or aminoglycoside nucleotidyltransferases (ANT or AAD), depending on their specific functions [55]. We only found aminoglycoside O-phosphotransferase (*APH(3′)-Ia* (*n*=9/27), *APH(3″)-Ib* (*n*=11/27) and *APH(6)-Id*) (*n*=11/27) in hospital isolates (none of the community isolates). Aminoglycoside nucleotidyltransferases *ANT(3″)-IIa*, intrinsic in this species, were found in all the isolates (*n*=27). Another major resistance mechanism to aminoglycoside is the acquisition of a 16S methyltransferase (*armA*), which confers broad-spectrum resistance to all clinically relevant drugs in this class of antibiotics, was also only found in the hospital strains (*n*=10/27) [56]. Three isolates (H-11699, C-72 and C-65) showed amikacin resistance despite the lack of the *armA* gene. The reasons for this would need further investigation. The sulfonamide resistance dihydropteroate synthase gene *sul2* was found in three hospital strains. However, *tetB* was the most frequently identified gene among MDR hospital strains, followed by macrolide resistance genes (*mphE* and *msrE*) (*n*=12/15), which conforms with the phenotype data.

Antimicrobial resistance in *A. baumannii* has been associated with four families of efflux pumps: the resistance nodulation division (RND) family, the major facilitator superfamily (MFS) family, the multidrug and toxic compound extrusion (MATE) family, and the small multidrug resistance (SMR) family [57]. The a*deABC* and RND-type efflux pumps are not only associated with aminoglycoside resistance but also with resistance to tigecycline lactams, chloramphenicol, erythromycin and tetracycline [58]. The complete *adeABC* package was detected in 12 hospital and 1 community *A. baumannii* isolates, with another 10 isolates carrying either 1 or 2 genes. Other RND-type efflux pumps, including a*deFGH* and a*deIJK*, which can contribute to multidrug resistance in *A. baumannii *, were commonly found in all 27 isolates. Apart from this, MFS efflux pumps, a*baQ* (*n*=23) and a*mvA* (*n*=27) mediate resistance to different types of antibiotics, including fluoroquinolones and macrolides [59, 60]. We found the SMR efflux pump-related gene a*beS* in 25 isolates, whereas a MATE family pump, *abeM*, was present in all *A. baumannii* isolates in our study. The chloramphenicol exporter gene, *flor*, was detected in a hospital strain, H-11553.

#### Virulence factors

**Fig. 3.**
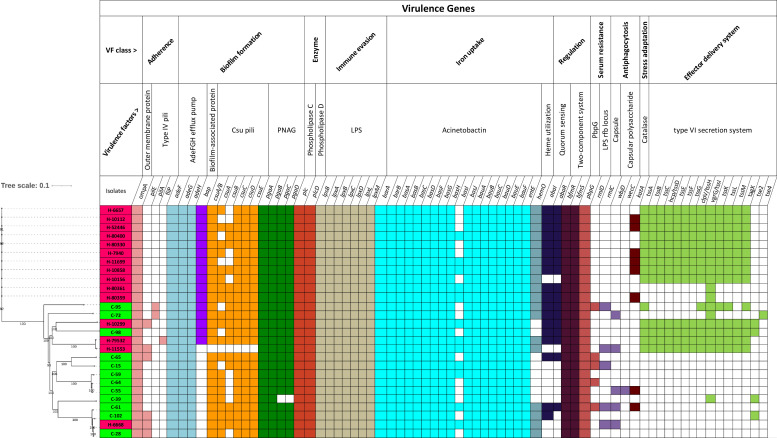
Presence of genes involved in virulence in the community and hospital *A. baumannii* genomes. The names of the isolates are shown on the left of the figure and are colour coded, as shown in Fig. 2. The isolates were organized here along with a phylogenetic tree (left) constructed by whole-genome SNP analysis. The presence of genes in an isolate is specified by a coloured rectangle, with different genes being coloured differently according to their mechanistic traits. The absence of genes is shown as white rectangles. These genes are grouped in the figure according to the mechanistic trait they are predicted to impart to *A. baumannii* strains.

All sequenced strains were examined for genes encoding virulence factors selected from the virulence factor database (VFDB). The results are summarized in Fig. 3. Similar virulence genes were present in most community and hospital isolates, which suggests the pathogenic nature of most strains in the current study.

However, there was a clear difference in the presence of genes encoding the type VI secretion system, which plays a vital role in the virulence of *A. baumannii *. The set of genes are present in 13 isolates, with another 5 isolates carrying some genes of this system [61]. Strikingly, the complete set of these genes, including core tss (*tssA-M*) and tag, was only found in 12 hospital isolates (not found in H-6668, H-80359 and H-80361) and 1 community isolate (C-98). A chi-square test revealed significant associations with presence in hospital strains and absence in community strains for 13 of the 15 genes tested that make up the type VI secretion system (*P*<0.05) (Table S4). The only genes not associated are *tse2* and *tse4*. Additionally, the gene encoding the biofilm-associated protein (*bap*), which acts in biofilm formation [62, 63], was found in 13 hospital and 3 community strains. This gene is complex in nature, having a long coding sequence comprising a variable number of repetitive regions [64].

Other virulence genes did not show any significant difference in frequency between hospital and community strains. To persist in iron-limited host habitats, *A. baumannii* develops high-affinity iron acquisition mechanisms, such as the siderophore acinetobactin [65]. Acinetobactin gene clusters (*barAB, basA-J, bauA-F, entE*) were present in almost all community and hospital isolates in this study, with the single exception that *basI* was not present in 15 isolates. Genes involved in biofilm and pili formation, adherence, quorum sensing, lipid A biosynthesis, phospholipase, two-component regulator systems and serum resistance were also detected in most of the *A. baumannii* isolates. The operon encoding the csu pili chaperone–usher assembly system that contributes to biofilm formation [66] and the *pgaABCD* operon, required for intercellular adhesin synthesis, were present in most of the isolates. *OmpA*, which codes for an outer-membrane protein, a key virulence factor that mediates bacterial biofilm formation, eukaryotic cell infection, antibiotic resistance and immunomodulation [67], was present in all isolates. The serum resistance gene *pbpG* (encoding penicillin-binding protein) was also found in all of our isolates.

#### Mobile genetic elements (MGEs)

High genetic plasticity in *A. baumannii* enables the accumulation of resistance determinants and the horizontal transfer of resistance genes through MGEs [68]. The presence of known MGEs, including transposons and insertion sequences in the sequenced community and hospital strains (shown in File S2; MGEs), was investigated. A search for composite transposons revealed that carbapenem resistance gene *bla*_OXA23_ was present inside *Tn2007* in all strains that carry the gene. Another strain, H-11553, carried *bla*_OXA-58_ within a composite transposon of *ISPssp2* (IS1 family). Apart from this, the transposon Tn*6080* surrounding target site duplications was found to carry the *bla*_OXA-51_-like gene in all 27 isolates.

The current study also found 16 insertion sequences (ISs) from 8 different families: IS3, IS5, IS6, IS8, IS30, IS66, IS91 and IS256. Some were widely distributed among the genomes investigated, such as IS17, while others were restricted to a single isolate (e.g. *ISAba10*, *ISAba49*, *ISAba27*, *ISEc29*).

#### The CRISPR arrays of community and hospital isolates were found to differ considerably

Out of 27 sequenced strains in this study, CRISPR arrays were detected in 8 community and 2 hospital strains (Table S6). Two CRISPR arrays were detected in three community strains (C-28, C-39, C-61) and one hospital strain (H-79532). No CRISPR arrays were present in any MDR strain. Antibiotic-susceptible strains or those with a lower number of resistance genes had higher instances of CRISPR. The arrays exhibited a series of repeated sequences and spacers associated with type I-F CRISPR systems.

We found identical spacer and repeat sequences between community strain C-61 and hospital strain H-6668. All 20 spacers of the C-61 CRISPR2 array were identical to the spacers of H-6668. This suggests a very close ancestral history between these isolates, consistent with their close placement in the phylogram in Fig. 2. We also detected identical spacers among two community strains, C-61 and C-28. However, no significant association was found between the CRISPR array and antibiotic resistance in our 27 isolates. (*P*-value >0.05).

#### Pangenome analysis

**Fig. 4.**
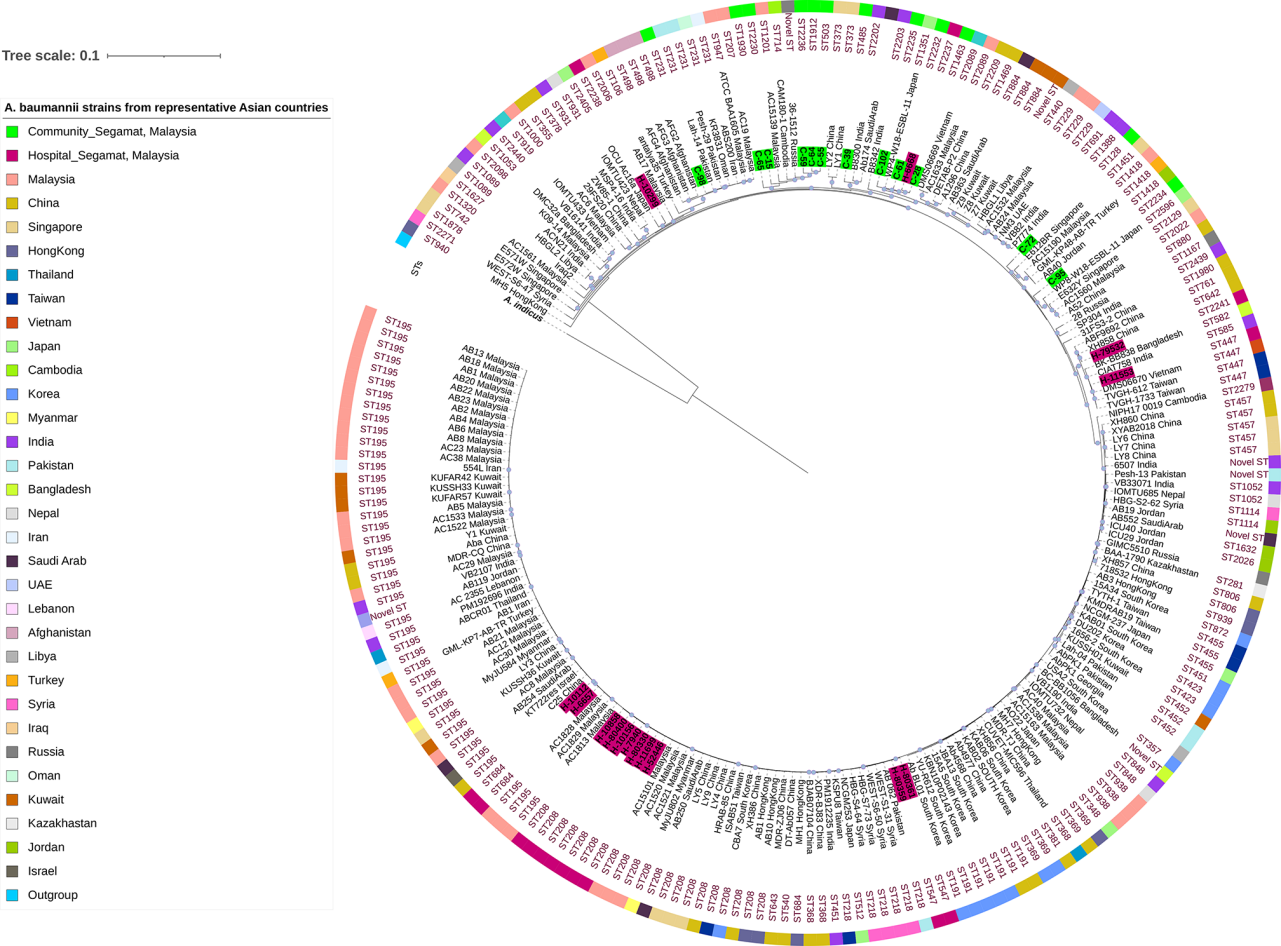
Core-genome-derived phylogenetic tree from pangenomes of 191 *A *. *baumannii* strains from Asian countries retrieved from the NCBI and the 27 *A *. *baumannii* strains from the current study. Terminal branches are labelled with the strain name and representative country names. Sequence types (STs) determined using the Oxford MLST scheme. Samples characterized in this study are shown in purple (hospital strains) and green (community strains). The blue circle symbols near the branches indicate bootstrap values of 100,

The functional adaptability of bacterial species can be better understood by extensive analysis of their pan-genome [69]. This analysis helps to identify core and accessory genes in the genome of *A. baumannii* species that might contribute to finding an ancestral relationship based on their genomic variation [70]. To assess the ancestral relationship of *A. baumannii* strains circulating in the Asian region, the gene content of the 27 genomes in this study was compared with another 191 *A *. *baumannii* sequences from Asian countries retrieved from the National Center for Biotechnology Information (NCBI) database (File S2; *A. baumannii* genomes from NCBI). The pangenome comprised a total of 22 885 genes, of which 18 682 (81.63 %), 1966 (8.59 %), 1481 (6.47 %) and 756 (3.30 %) were identified as cloud, shell, soft-core and core genes shared among the 218 isolates. Cloud (0 %<=strains<15 %), shell (15 %<=strains<95 %) and soft-core genes (95 %<=strains<99 %) are the accessory genes (gene set shared within one or some strains) as opposed to core genes (99 %<=strains<=100 %) that are shared by almost all clade members [71]. In these isolates, the proportion of the pan-genome-containing genes related to transcriptional regulators and transporters was 3.15 % (721) and 3.70 % (847), respectively. Moreover, we discovered genes that code for transposases and bacteriophage proteins (heads, tails and capsids) in 3.39 % (727) and 1.52 % (327) of the accessory genome, respectively. Genes encoding hypothetical proteins were found in 0.61 % (140) of core genomes and 45.64 % (10 446) of accessory genomes. A core gene-based phylogenetic tree was created from the pangenome, which reflects the relationships between the genomes of our study and selected other genomes (Fig. 4). The phylogenetic tree could be divided into numerous clades based on STs. *Acinetobacter indicus* was used as an outgroup. We observed a similar clustering pattern to that of the SNP-based phylogeny. The hospital strain H-6668 clustered with C-28, C-61 and C-102, consistent with the SNP phylogeny, and again indicating a close relationship between these isolates. Similar clustering was also observed between C-98 and H-10299 in the SNP phylogeny, as we found them in the same branch in pangenome phylogeny. When we made a comparison with other Asian countries, 10 hospital and 2 community isolates from our study clustered with other Malaysian isolates. The remaining 15 hospital and community isolates were clustered with isolates from PR China, Japan, the Republic of Korea, Vietnam, India, Bangladesh, Singapore, the Russian Federation and Afghanistan.

## Discussion

*A. baumannii* is one of the most frequently encountered pathogens in human infections and is recognized as a significant reservoir of MDR genes [72]. Most of the previous studies have focused on *A. baumannii* isolated from hospitals and only a few studies have focused on isolates from the community or environment [73, 74]. Ours is the first study investigating the epidemiology of *A. baumannii* recovered from the human gut as a potential reservoir in the community and from a tertiary care hospital located in the same district to establish whether there is possible transmission between these two sources. This is of crucial importance in the field of epidemiology due to *A. baumannii* being a priority pathogen with scarce data on community carriage, especially in healthy individuals in Southeast Asia [75].

The isolation rate of *A. baumannii* from the human faecal samples in the Segamat community was 3.9 % (*n*=9/233) and 2.4 % (*n*=3/126) in 2018 and 2019, respectively, which is consistent with prevalence rates from faecal samples reported in Senegal 5.4 % (*n*=39/774), the UK and the Netherlands (2/226) [74, 75]. It is possible that a more exhaustive analysis of individual faecal samples would have revealed greater carriage rates, but that was not possible due to our large sample sizes. Several studies have also focused on human skin as a community reservoir of *A. baumannii *. Various studies have found the prevalence of *A. baumannii* on human skin to be 0.5, 2.5 % or much higher [74, 76, 77]. Thus, despite *A. baumannii* not being widespread in non-hospitalized individuals, it can be, and has been, recovered at low frequencies from the skin and faecal flora. The 12 isolates from the community in our study appear not to be commensal. However, these organisms could act as potential reservoirs of genes and transfer antibiotic resistance genes horizontally to other bacterial species in the human gut and impact on community health during their carriage in the gut. Besides community isolates, we obtained 15 *A *. *baumannii* isolates from hospitalized individuals and the majority were from tracheal aspirates (7/15).

A comparison of antibiotic resistance profiles between community and hospital isolates revealed that the hospital isolates had an elevated level of resistance. Most community isolates were either susceptible or showed intermediate resistance to the antibiotics tested. Our study found colistin- and polymyxin B-resistant hospital strains. These two antibiotics have become the last-line therapy for antibiotic-resistant *A. baumannii* infections [78]. Moreover, 80 % of the hospital isolates from Segamat were highly resistant to carbapenem (imipenem and meropenem). Malaysia’s National Surveillance on Antibiotic Resistance (NSAR) reported that countrywide carbapenem resistance rates were ~50–60 % from 2008 to 2016 [79], and even higher rates have been reported in the university hospital in Kuala Lumpur [80]. These studies, along with ours, suggest that carbapenem-resistant *A. baumannii* strains are widely distributed in hospital settings, with a risk of spreading into the community.

Considerable differences were found between the MDR and non-MDR phenotype and genotype data among the community and hospital *A. baumannii* isolates, which were expected, as the hospital is known to be a hotspot for MDR bacteria. AMR gene analysis based on whole-genome sequences revealed that all strains carried beta-lactam resistance genes (OXA51*, bla*_ADC_). However, most community and three hospital strains were sensitive to beta-lactam antibiotics. We have found several point mutations in beta-lactamase genes in non-resistant isolates leading to non-conservative amino acid changes. These changes may lead to lower functionality of the protein products. However, this needs further testing. In addition, the carbapenem resistance OXA-23 and OXA-24 like genes were identified in 11 and 1 hospital strains, respectively, confirming our AST results indicating that these strains were resistant to carbapenems. Apart from AMR, WGS revealed several virulence factors that were found in all 27 sequenced strains. Genes coding for the type VI secretion system (T6SS), which is known as a major virulence factor in *A. baumannii *, were found in most of the hospital strains and one community strain. T6SS genes can be exploited to produce toxins that kill other bacteria and even eukaryotic cells [81].

MGEs play an essential role in regulating and disseminating antimicrobial resistance genes [64]. Composite transposons (Tn2006, Tn2007, Tn2008 and Tn2009) flanked by two copies of similar insertion sequence elements are associated with the transfer of *bla*_OXA23_ in *A. baumannii* [82]. Our study also found the presence of OXA23 genes within Tn2007 in MDR hospital strains. Likewise, transposons and different insertion sequences were widely distributed among community strains. The role of MGEs in genetic exchange between hospital and community strains was not explored.

The CRISPR–Cas mechanism is considered to be an adaptive immune system based on identifying past infections in prokaryotes. According to CRISPRCasdb [83], roughly 20 % of genus *Acinetobacter* representatives and 18 % of isolates of *A. baumannii* species possess both CRISPR arrays and Cas genes [84]. The current analysis discovered putative CRISPR–Cas systems in eight community and just two hospital strains, and all of these isolates were susceptible to antibiotics. Isolates lacking CRISPR arrays and active Cas genes were demonstrated to contain considerably more antibiotic resistance genes than those lacking either or both, although the numbers analysed were not sufficient to produce a statistically significant result. These results suggest a link between the susceptible and resistant genotype/phenotypic strain of *A. baumannii* and the type of its CRISPR–Cas system. Thus, CRISPR–Cas systems could also play a critical role in controlling the expression of many genes that determine the resistance pattern and pathogenicity of isolates and the propagation of antibiotic resistance genes in *A. baumannii* [84, 85].

Comparative whole-genome sequence analysis of the community and hospital *A. baumannii* strains revealed the possible ancestral relationship of some isolates within these settings. Nearly similar clustering was observed in both phylogenetic trees after whole-genome SNP and pangenome-based core gene analysis. The drug-susceptible isolates in this study were distinct from the MDR strains and there was apparent clustering between the hospital isolate H-6668 and the community strains C-28, C-61 and C-102. Furthermore, C-61 and H-6668 had identical CRISPR arrays. All of these isolates were found to be antibiotic-susceptible. We also observed another cluster between H-10299 and C-98 after pangenome and SNP core genome phylogeny. There was also one cluster containing MDR strain H-11553 and non-MDR strain H-79532. Both of the strains carried similar beta-lactam resistance genes with certain mutations (ADC-76 and OXA-66). They also carried similar type VI secretion-related genes.

These observations are consistent with previous reports of transmission from patient to patient, patient to health care worker, patient to the environment and other related sources [86]. The results demonstrate that there could have been a common ancestor of some of these hospital and community *A. baumannii* isolates if we disregard the possibility of adventitious isolation.

## Conclusion

This study characterized the variation between *A. baumannii* strains isolated from two different sources in a district in Malaysia as a pilot study. We found that the human gut could potentially act as a reservoir for *A. baumannii* colonization, even though carriage in the same individuals was not detected after a year. Hospitals have been identified as the primary source of MDR *A. baumannii* in Malaysia. Less drug resistance was demonstrated by community isolates, indicating that higher antibiotic selection pressure occurred in the hospital settings. Comparative WGS results of *A. baumannii* strains revealed the possible ancestral relationship between some isolates, suggesting the chances of a single origin for some of the strains in these two different settings. This study also suggests that the community strains could easily turn into pathogenic strains if they gain resistance genes via horizontal gene transfer, perhaps by losing the CRISPR loci. Studies focusing on a larger sample of *A. baumannii* isolates from the community, hospitals and surrounding environments in different geographical locations would improve our understanding of this relationship. This study sheds light on the public health concerns associated with the dissemination of *A. baumannii* in a healthy community and may aid in devising public health measures to limit the spread of this pathogen in the future.

## Supplementary Data

Supplementary material 1

Supplementary material 2
