# Supplementary material for: Molecular characterization and comparative genomic analysis of Acinetobacter baumannii isolated from the community and the hospital: an epidemiological study in Segamat, Malaysia
Source: Microb Genom. 2023 Apr 5;9(4):mgen000977. doi: 10.1099/mgen.0.000977 (PMC10210948; doi:10.1099/mgen.0.000977)
Supplement: Supplementary material 1 [file mgen-9-977-s001.pdf]

Supplementary Material_S1

Molecular characterization and comparative genomic analysis of *Acinetobacter baumannii* isolated from the community and the hospital: an epidemiological study in Segamat, Malaysia

Nazmul Hasan Muzahid[1,*], Md Hamed Hussain[1], Marie Andrea Laetitia Huët [1], Jacky Dwiyanto[1], Tin Tin Su[2], Daniel Reidpath[2], Faizah Mustapha[3], Qasim Ayub[1,4,5], Hock Siew Tan[1,5] and Sadequr Rahman[1,5,*]

[1]School of Science, Monash University Malaysia, 47500, Bandar Sunway, Selangor Darul Ehsan, Malaysia

[2]South East Asia Community Observatory (SEACO) and Global Public Health, Jeffrey Cheah School of Medicine and Health Sciences, Monash University Malaysia, Bandar Sunway, 47500, Subang Jaya, Selangor, Malaysia

[3]Department of Pathology, Hospital Segamat, Jalan Genuang, Bandar Putra, 85000, Segamat, Johor, Malaysia

[4]Monash University Malaysia Genomics Facility, 47500, Bandar Sunway, Selangor Darul Ehsan, Malaysia

[5]Tropical Medicine and Biology Multidisciplinary Platform, Monash University Malaysia, Bandar Sunway, 47500, Subang Jaya, Selangor, Malaysia.

Corresponding author

Nazmul Hasan Muzahid; nazmul.muzahid@monash.edu
Sadequr Rahman; sadequr.rahman@monash.edu

Table S1: Details of *A. baumannii* isolates from the Segamat hospital, Malaysia

SL No.	Strain ID	Patient Age	Gender	Diagnosis	Isolation source
1	H-AB-6657	78	M	Severe pneumoniae	-
2	H-AB-6668	63	M	Community acquired pneumonia	Blood
3	H-AB-7940	40	F	Deep vein thrombosis	Blood
4	H-AB-10112	87	F	Ventilator associate pneumonia	tracheal aspirate
5	H-AB-10156	73	M	Invasive Klebsiella Pneumonia Syndrome	Sputum CRS
6	H-AB-10299	79	M	Septic shock	tracheal aspirate
7	H-AB-10858	55	F	Septic shock	tracheal aspirate
8	H-AB-11553	75	M	community acquired pneumonia	tracheal aspirate
9	H-AB-11699	50	M	CRBSI	blood
10	H-AB-79532	28	M	Traumatic Brain Injury	tracheal aspirate
11	H-AB-52446	39	M		Urine
12	H-AB-80330	43	M	Severe leptospirosis	Tracheal aspirate
13	H-AB-80359	44	M	Cryptococcal meningitis	Blood
14	H-AB-80400	62	M	Pneumonia	Tracheal aspirate
15	H-AB-80361	44	M	Cryptococcal meningitis	Blood

Table S2: Details of *A. baumannii* isolates from the community in Segamat, Malaysia

Sample Collection Year	Stoolcode	Household	Location	Relatio-nship	Gender	Age	Ethnicity	Occupation	Chronic diabetes
2018	C-15	8	Chaah	Head	Male	62	Indian	agricultural	Yes
2018	C-28	17	Chaah	Wife	Female	50	Indian	homemaker	No
2018	C-55	38	Bekok	Wife	Female	33	Jakun	homemaker	No
2018	C-61	35	Bekok	Head	Male	39	Chinese	craft	No
2018	C-65	44	Chaah	Head	Male	69	Indian	unemployed	Yes
2018	C-72	103	Chaah	Head	Female	54	Indian	service	Yes
2018	C-95	113	Chaah	Head	Male	36	Indian	service	No
2018	C-98	125	Chaah	Wife	Female	46	Indian	homemaker	No
2018	C-102	33	Chaah	Head	Male	54	Indian	elementary	No
2019	C-39	190	Bekok	Wife	female	46	Jakun	homemaker	No
2019	C-59	108	Chaah	Head	Male	52	Indian	unemployed	Yes
2019	C-64	113	Chaah	Head	Male	37	Indian	service	No

Table S3: MLST Profiles of *A. baumannii* hospital and community Isolates

SL	Isolates	ST	CC	International Clone
1	H-7940	208	208/92	IC-2
2	H-10156	208	208/92	IC-2
3	H-10858	208	208/92	IC-2
4	H-11699	208	208/92	IC-2
5	H-52446	208	208/92	IC-2
6	H-80330	208	208/92	IC-2
7	H-80400	208	208/92	IC-2
8	H-11553	447	447	IC-8
9	H-80359	547	208/92	IC-2
10	H-80361	547	208/92	IC-2
11	H-79532	2241	642	ND
12	H-6657	684	208/92	IC-2
13	H-10112	684	208/92	IC-2
14	H-6668	2237	ND	ND
15	H-10299	2238	ND	ND
16	C-28	1463	ND	ND
17	C-65	1930	1108	ND
18	C-98	231	231	IC-1
19	C-64	1912	ND	ND
20	C-39	485	1138	ND
21	C-15	2230	ND	ND
22	C-55	503	1171	ND
23	C-61	2232	953	ND
24	C-72	128	ND	IC-2
25	C-95	2234	ND	ND
26	C-102	2235	ND	ND
27	C-59	2236	ND	ND

NB; SL 1-15; Hospital isolates, SL 16-27; Community isolates, ST; Sequence Type, CC; Clonal complex, IC; International clone, ND; Not determined

Table S4: Results from chi-square test for the presence and absence of type VI secretion system (T6SS) genes in hospital and community isolates.

		Hospital		Community		Total	chi-squared P value
**Gene**	**Presence**	**n**	**%**	**n**	**%**	**N**	
tssA	Present	12	0.86	2	0.14	14	0.007
	Absent	3	0.23	10	0.77	13	0.05
tssB	Present	12	0.92	1	0.08	13	0.002
	Absent	3	0.21	11	0.79	14	0.03
tssC	Present	12	0.92	1	0.08	13	0.002
	Absent	3	0.21	11	0.79	14	0.03
hcp/tssD	Present	12	0.92	1	0.08	13	0.002
	Absent	3	0.21	11	0.79	14	0.03
tssE	Present	12	0.92	1	0.08	13	0.002
	Absent	3	0.21	11	0.79	14	0.03
tssF	Present	12	0.92	1	0.08	13	0.002
	Absent	3	0.21	11	0.79	14	0.03
tssG	Present	12	0.92	1	0.08	13	0.002
	Absent	3	0.21	11	0.79	14	0.03
clpV/tssH	Present	12	0.86	2	0.14	14	0.007
	Absent	3	0.23	10	0.77	13	0.05
vgrG/tssI	Present	14	0.78	4	0.22	18	0.02
	Absent	1	0.11	8	0.89	9	0.02
tssK	Present	12	0.86	2	0.14	14	0.007
	Absent	3	0.23	10	0.77	13	0.05
tssL	Present	12	0.86	2	0.14	14	0.007
	Absent	3	0.23	10	0.77	13	0.05
tssM	Present	12	0.92	1	0.08	13	0.002
	Absent	3	0.21	11	0.79	14	0.03
tagX	Present	12	0.86	2	0.14	14	0.007
	Absent	3	0.23	10	0.77	13	0.05
tse2	Present	1	0.25	3	0.75	4	0.3
	Absent	14	0.61	9	0.39	23	0.29
tse4	Present	0	0.00	1	1.00	1	NA
	Absent	15	0.58	11	0.42	26	0.43

Table S5: Multiple variants were observed between 27 sequenced hospital and community isolates with the reference strain *A. baumannii* AC30. Here, SNP stands for Single Nucleotide Polymorphism, MNP is Multiple Nucleotide Polymorphism, INS is Insertion, DEL is Deletion and Complex is the combination of snp/mnp.

Isolates	SNP	MNP	Ins	Del	Complex
**C-15**	36966	427	201	196	7131
**C-28**	36447	155	169	183	7414
**C-39**	35645	141	166	172	6758
**C-55**	35956	416	171	163	6773
**C-59**	36887	449	212	201	6938
**C-61**	36572	167	161	196	7202
**C-64**	37292	395	198	192	7134
**C-65**	35985	119	152	149	6776
**C-72**	38386	440	247	209	7770
**C-95**	35398	335	158	152	6434
**C-98**	37745	140	151	150	7489
**C-102**	36205	145	175	222	7174
**H-6657**	653	3	4	9	187
**H-6668**	37320	1622	194	207	7492
**H-7940**	466	5	8	7	151
**H-10112**	452	0	2	8	135
**H-10156**	478	6	7	7	161
**H-10299**	37773	128	193	182	7418
**H-10858**	469	9	8	8	148
**H-11553**	37619	126	184	186	7633
**H-11699**	447	2	6	5	144
**H-52446**	504	8	9	3	175
**H-79532**	39120	400	223	227	7822
**H-80330**	476	8	8	6	148
**H-80359**	452	2	7	5	168
**H-80361**	448	7	6	5	159
**H-80400**	473	6	8	6	155

Table S6: Profiling of CRISPR-Cas loci among the community and hospital *A. baumannii* strains

Strains	Number of CRISPR Arrays	CRISPR repeat length	CRISPR length	Number of Spacers per Strain	Repeat sequence
**C-15**	ND	ND	ND	ND	ND
**C-28**	2	28, 28	9096, 327	151, 5	TTTCTAAATGGCGTATGCCGCCATGAAC
**C-39**	2	28, 28	2429, 4583	40, 76	GTTCATGGCGGCATACGCCATTTAGAAA/ TTTCTAAATCATCTATGCGATGAAGAAC
**C-55**	ND	ND	ND	ND	ND
**C-59**	1	28	447	7	TTTCTAAGCTGCCTATACGGCAGTGAAC
**C-61**	2	28, 28	5129, 1232	85, 20	TTTCTAAATGGCGTATGCCGCCATGAAC/ GTTCATGGCGGCATACGCCATTTAGAAA
**C-64**	ND	ND	ND	ND	ND
**C-65**	ND	ND	ND	ND	ND
**C-72**	1	28	1167	19	GTTCGTCATCGCCCAGATGATTTAGAAA
**C-95**	1	28	3510	58	GTTCATGGCGGCATACGCCATTTAGAAA
**C-98**	1	28	3449	57	GTTCATGGCGGCATACGCCATTTAGAAA
**C-102**	1	28	3813	63	TTTCTAAATGGCGTATGCCGCCATGAAC
**H-6657**	ND	ND	ND	ND	ND
**H-6668**	1	28	8676	144	GTTCATGGCGGCATACGCCATTTAGAAA
**H-7940**	ND	ND	ND	ND	ND
**H-10112**	ND	ND	ND	ND	ND
**H-10156**	ND	ND	ND	ND	ND
**H-10858**	ND	ND	ND	ND	ND
**H-10299**	ND	ND	ND	ND	ND
**H-11553**	ND	ND	ND	ND	ND
**H-11699**	ND	ND	ND	ND	ND
**H-52446**	ND	ND	ND	ND	ND
**H-79532**	2	28, 28	4547, 865	75, 14	TTTCTAAATCATCTGGGCGATGACGAAC, GTTGTTCATCGCATAGATGATTTAGAAA
**H-80330**	ND	ND	ND	ND	ND
**H-80359**	ND	ND	ND	ND	ND
**H-80361**	ND	ND	ND	ND	ND
**H-80400**	ND	ND	ND	ND	ND

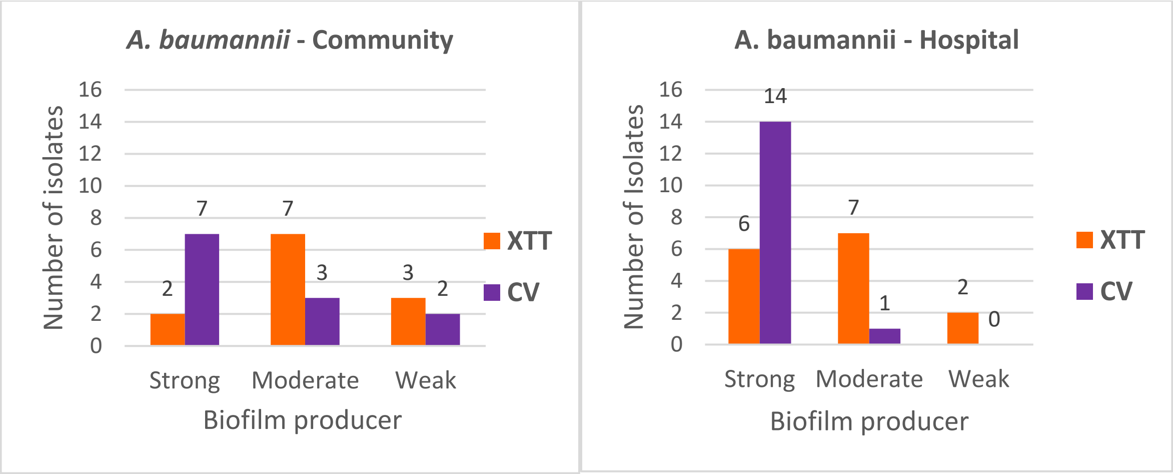

Fig. S1: Biofilm-forming ability of the community (n = 12) and hospital (n = 15) *A. baumannii* isolates. The biofilm producers were classified into 4 groups consisting of strong, moderate, weak, and non-biofilm producers according to [38].

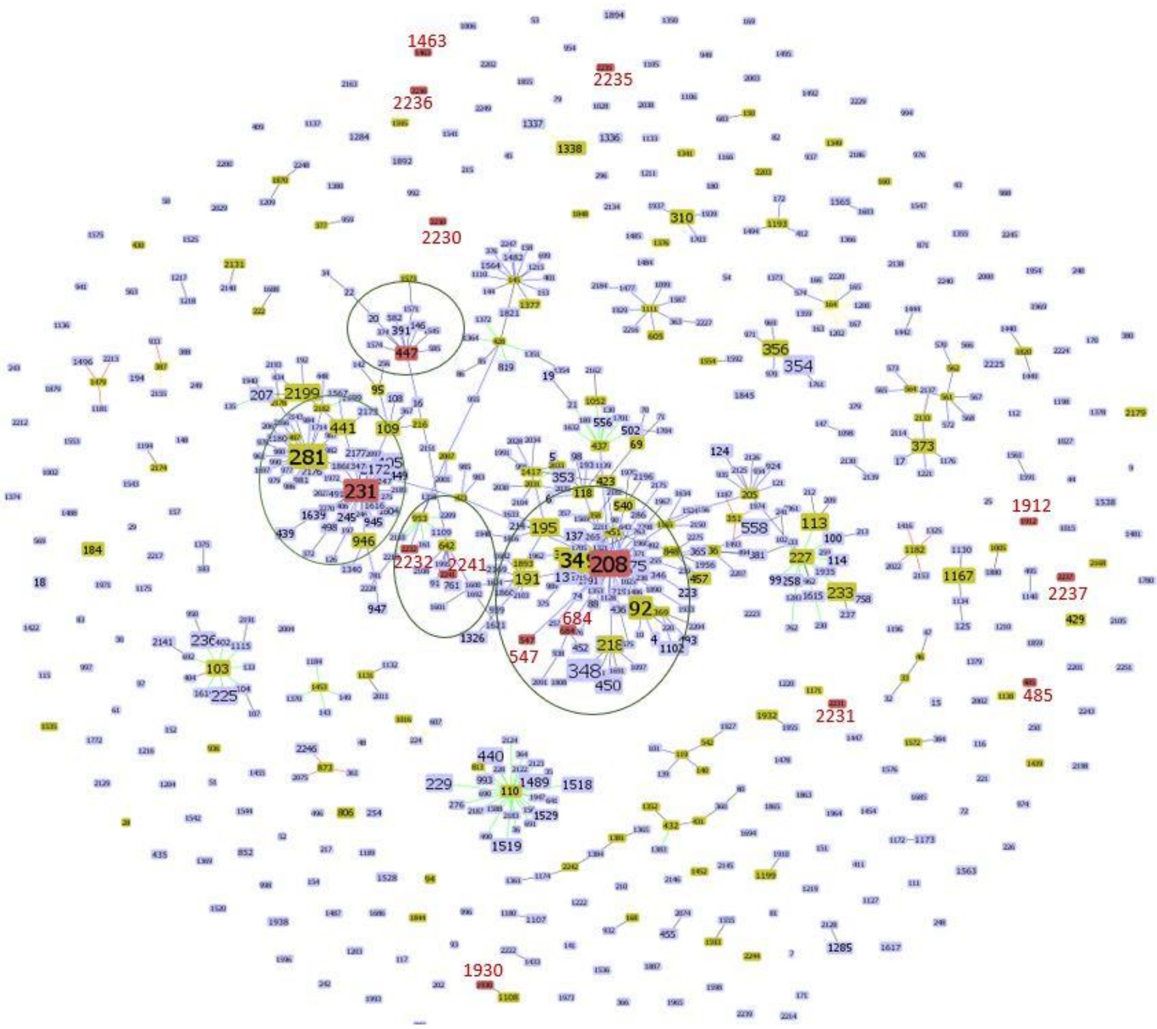

Fig. S2 Population snapshot. goeBURST analysis of 2275 ST present in the PubMLST database. The red colour STs annotated here are from the present study. The study's largest CCs are marked with a green circle (CC-208, CC-231, CC-447, CC-642 and CC-953. The snapshots here were created with groups defined at a TLV (Triple locus variant) level.

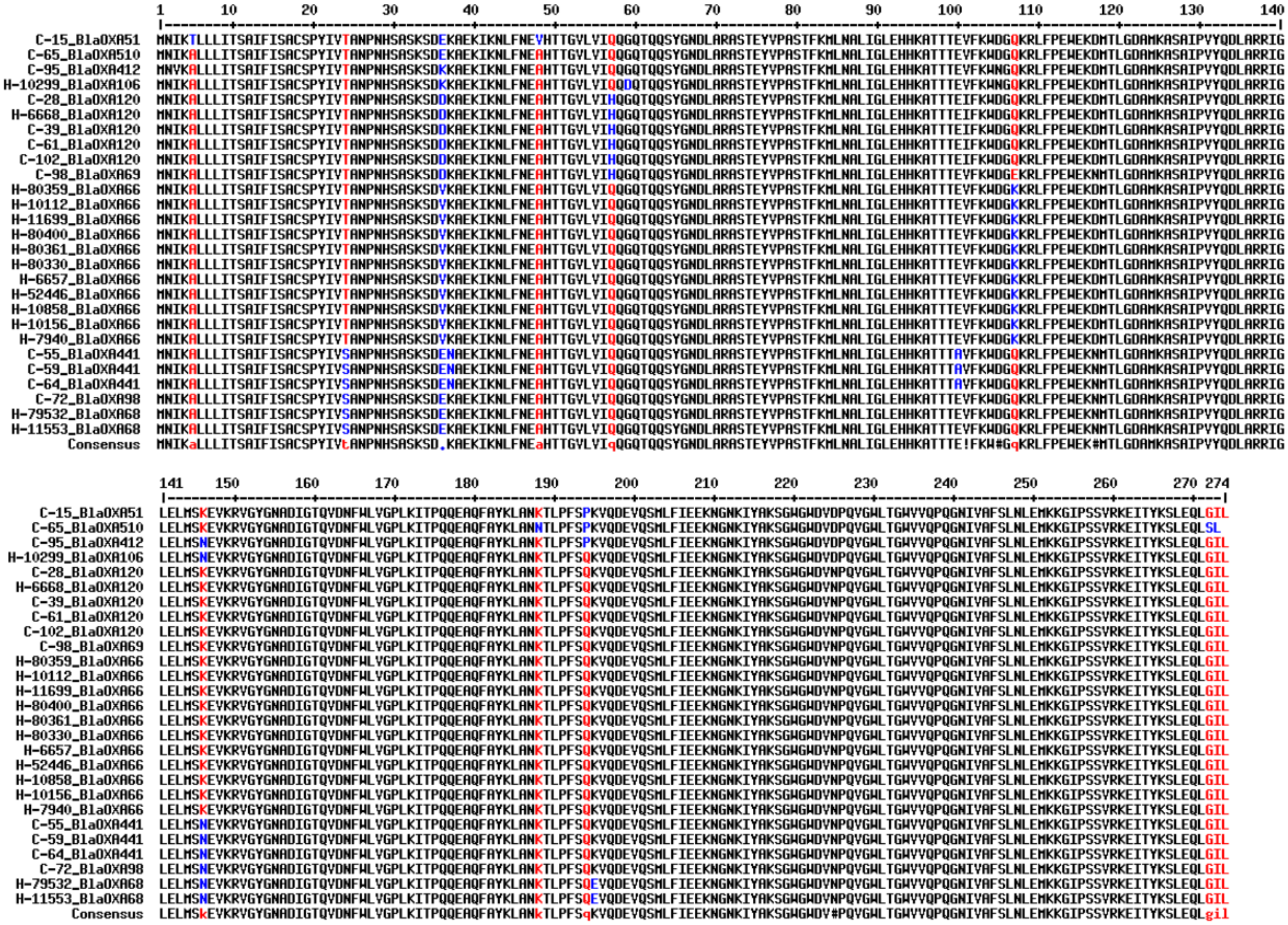

Fig S3: Multiple sequence alignment of translated OXA-51 type gene of 27 hospital and community *A. baumannii* isolates. Here black color sequences showing the high consensus alignment. Red color reparents low consesnsus and blue indicate neutral consensus.

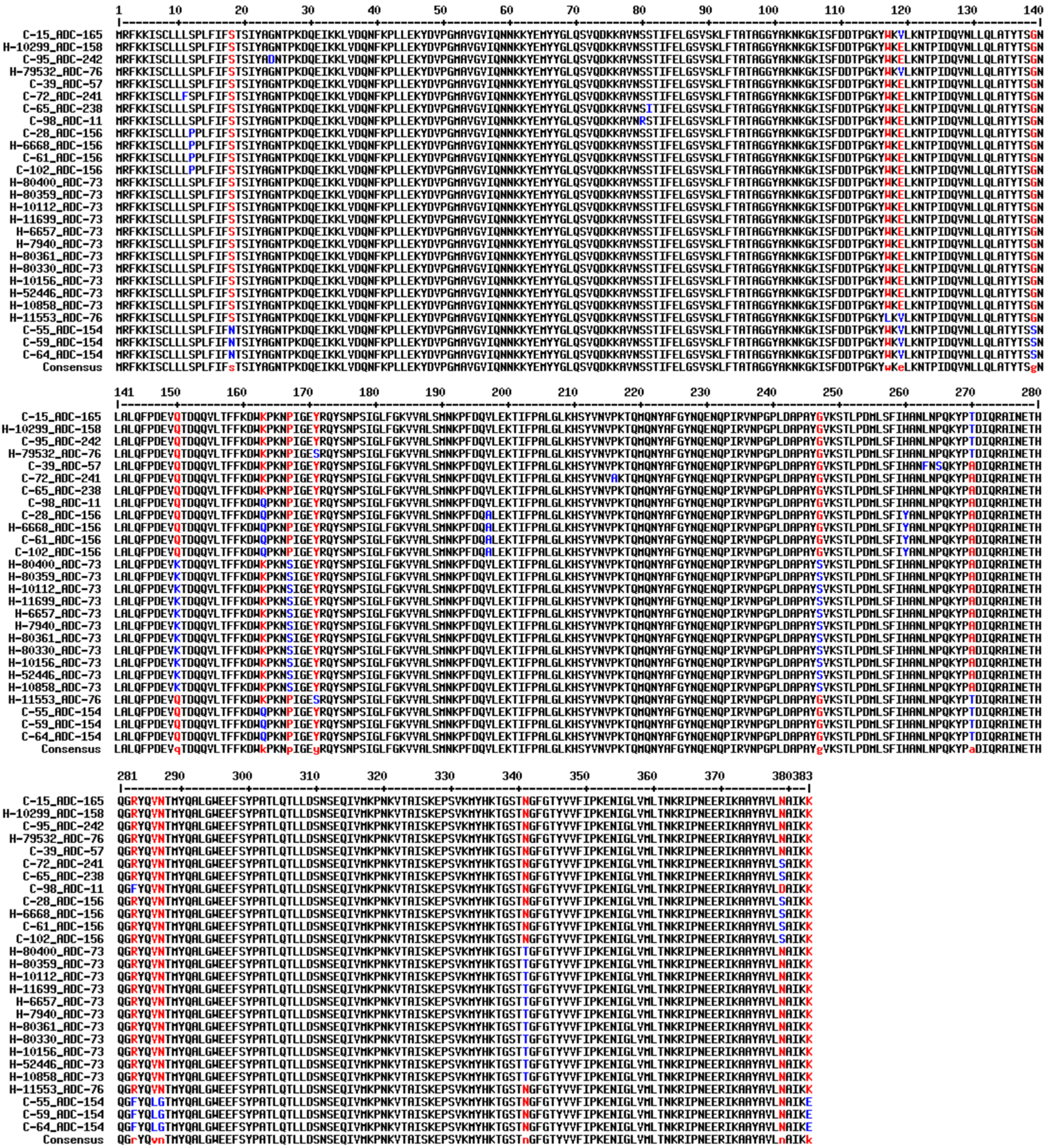

Fig S4: Multiple sequence alignment of translated class C beta lactamase ADC type gene of 27 hospital and community *A. baumannii* isolates. Here black color sequences showing the high consensus alignment. Red color reparents low consesnsus and blue indicate neutral consensus.

Supplementary text S1

Experimental Methods:

DNA extraction using heat boiling method

In details, 2 to 3 colonies from fresh culture were transferred in a sterile PCR tube containing 50 µL of sterile ultrapure water. The suspension was then boiled at 95°C for 5 minutes to allow cell lysis and extraction of DNA. After that, the PCR tubes were placed on ice for 5 minutes followed by centrifugation at 16000 *g* for 5 minutes. The supernatant containing the bacterial DNA was then transferred to a new sterile PCR tube.

Table S7: Primers used in this study

Gene Targeted	Primer	Primer Sequences (5′→3′)	Expected Amplicon Size
16s rRNA	27F	TACGGYTACCTTGTTACGACTT	1465
	1492R	AGAGTTTGATCMTGGCTCAG	
gyrB	Sp2F	GTTCCTGATCCGAAATTCTCG	490
	Sp4R	AACGGAGCTTGTCAGGGTTA	

PCR Amplification and Sequencing of 16S rRNA Gene

Table S8: Reagent conditions for 16S rRNA PCR assay

Reagent	Initial Concentration	Final Concentration	Volume per reaction/µl
MyTaq Red Mix (Bioline)	2x	1x	25

27F (Forward primer)	10 µM	0.4 µM	2
1492R (Reverse primer)	10 µM	0.4 µM	2
DNA Template	-	-	5
Water (ddH20)	-	-	16
**Total Volume**	-	-	50

Table S9: Reaction conditions for 16S rRNA PCR assay

Amplification step	Temperature	Time
Initial Denaturation	95°C	10min
30 cycles	95°C	1min
	55°C	1min
	72°C	1min 30s
Final Elongation step	72°C	15min

Screening of PCR products

All 16S rRNA PCR amplicon products were subjected to electrophoresis using 1.2% agarose gel along with a 1 kb DNA ladder (Gene Ruler, Thermo Scientific). The agarose gel was run at 80V for 40 minutes and visualised using SafeView™ DNA Stain (ABM) and GelDoc™ EZ Gel Documentation System (Bio-Rad). After that, PCR products were sent to First BASE DNA Sequencing Singapore (https://order.base-asia.com/) for Sanger sequencing

Detection of *Acinetobacter baumannii* using Species-specific *gyrB* gene

Table S10: Reagent conditions for *A. baumannii* using Species-specific *gyrB* gene PCR assay

Reagent	Initial Concentration	Final Concentration	Volume per reaction/µl
MyTaq Red Mix (Bioline)	2x	1x	12.5
Sp2F (Forward primer)	10 µM	0.4 µM	0.5
Sp4R (Reverse primer)	10 µM	0.4 µM	0.5
DNA Template	-	-	1
Water (ddH20)	-	-	10.5
**Total Volume**			25

Table S11: Reaction conditions for *A. baumannii* using Species-specific gyrB gene PCR assay

Amplification step	Temperature	Time
Initial Denaturation	94°C	2min
25 cycles	94°C	1min
	60°C	30s
	72°C	1min
Final Elongation step	72°C	10min

Biofilm Formation Assay

100 µl of TSB medium supplemented with 0.2% glucose was added to each well. Using overnight bacterial culture, the cell suspension was adjusted to 0.5 McFarland standard in TSB supplemented with 0.2% glucose. 100 µl of each suspension was inoculated to each well. The microplates were incubated at 37°C for 20 hours for biofilm production.

XTT reduction assay

XTT assay was performed following the method used by (Lin et al., 2020) with some modifications. Fresh XTT solution was made by dissolving 1.5 mg XTT (Sigma) in 1.5 ml prewarmed (37 °C) physiological saline before each assay. The solution was filter- sterilized and then supplemented with 0.4mM of 300 µl menadione solution, containing 1.72 mg menadione (Sigma) in 10 ml acetone. After production of biofilm, the wells were washed with PBS. In each well 12 µl of XTT-menadione solution and 200 µl of fresh TSB medium were added. Plates were incubated for one hour at 37 °C. After incubation, 100 µl of well contents transferred to a new microtiter plate and the absorbance was measured with a microplate reader (Tecan, Switzerland) at 490 nm. Negative control wells were included in the plate and *Staphylococcus aureus* ATCC 6538P which will give strong biofilm formation was used as the positive control. The assay was performed in three biological and technical replicates.

Crystal violet assay (CV assay)

Following the biofilm formation assay, after 24 hours of incubation at 37ºC, the bacteria culture was discarded. To remove the remaining bacteria cells the plate was washed twice with 200 µL of sterile phosphate-buffered saline followed by heat fixation at 60ºC for 1 hour. Adherent bacteria were stained with 200 uL of 0.1% Crystal violet for 20 mins. Excess stain was washed under running tap water. Subsequently, the microplates were vigorously tapped on napkins to remove any excess liquid. 200 µl of 95% ethanol was added in each well. Following a thirty minute period, 100 µl of well contents transferred to a new microtiter plate and the absorbance of each well was measured with a microplate reader (Tecan, Switzerland) at 595 nm. Negative control wells were included in the plate and *Staphylococcus aureus* ATCC 6538P which will give strong biofilm formation was used as the positive control. The assay was performed in three biological and technical replicates.

The average OD of the negative wells (with TSB only) was set as the cut-off value (ODc). Biofilm producers were classified into different categories according to their OD measurement (Zhang

et al., 2016) as non-biofilm producer (OD of isolates ≤ ODc), weak biofilm producer (ODc < O(2 × ODc < OD of isolates D of isolates ≤ 2 × ODc), moderate biofilm producer ≤ 4 × ODc) and strong biofilm producer (4 × ODc > OD of isolates).
